# *TaRECQ4* contributes to maintain both homologous and homoeologous recombination during wheat meiosis

**DOI:** 10.3389/fpls.2023.1342976

**Published:** 2024-01-29

**Authors:** Jeanne Bazile, Isabelle Nadaud, Pauline Lasserre-Zuber, Jonathan Kitt, Romain De Oliveira, Frédéric Choulet, Pierre Sourdille

**Affiliations:** ^1^ INRAE, UMR 1095 INRAE – UCA Genetics, Diversity & Ecophysiology of Cereals, Clermont-Ferrand, France; ^2^ Biotech Research and Innovation Centre (BRIC), Faculty of Health and Medical Sciences, University of Copenhagen, Copenhagen, Denmark

**Keywords:** meiosis, crossover, homologous recombination, homoeologous recombination, chiasmata, synapsis

## Abstract

**Introduction:**

Meiotic recombination (or crossover, CO) is essential for gamete fertility as well as for alleles and genes reshuffling that is at the heart of plant breeding. However, CO remains a limited event, which strongly hampers the rapid production of original and improved cultivars. *RecQ4* is a gene encoding a helicase protein that, when mutated, contributes to improve recombination rate in all species where it has been evaluated so far.

**Methods:**

In this study, we developed wheat (*Triticum aestivum* L.) triple mutant (TM) for the three homoeologous copies of *TaRecQ4* as well as mutants for two copies and heterozygous for the last one (Htz-A, Htz-B, Htz-D).

**Results:**

Phenotypic observation revealed a significant reduction of fertility and pollen viability in TM and Htz-B plants compared to wild type plants suggesting major defects during meiosis. Cytogenetic analyses of these plants showed that complete absence of *TaRecQ4* as observed in TM plants, leads to chromosome fragmentation during the pachytene stage, resulting in problems in the segregation of chromosomes during meiosis. Htz-A and Htz-D mutants had an almost normal meiotic progression indicating that both *TaRecQ4-A* and *TaRecQ4-D* copies are functional and that there is no dosage effect for *TaRecQ4* in bread wheat. On the contrary, the *TaRecQ4-B* copy seems knocked-out, probably because of a SNP leading to a Threonine>Alanine change at position 539 (T539A) of the protein, that occurs in the crucial helicase ATP bind/DEAD/ResIII domain which unwinds nucleic acids. Occurrence of numerous multivalents in TM plants suggests that *TaRecQ4* could also play a role in the control of homoeologous recombination.

**Discussion:**

These findings provide a foundation for further molecular investigations into wheat meiosis regulation to fully understand the underlying mechanisms of how *TaRecQ4* affects chiasma formation, as well as to identify ways to mitigate these defects and enhance both homologous and homoeologous recombination efficiency in wheat.

## Introduction

Meiosis is an essential process in eukaryotic reproduction, which entails a single round of DNA replication event followed by two successive rounds of chromosome segregation. This results in the production of haploid gametes that possess half the chromosome number of the initial diploid cell, the diploid state of the individual being restored upon fertilization (Mercier et al., 2015). In almost all eukaryotes, the faithful segregation of homologous chromosomes during meiosis relies on the prior formation of physical connections between them called “chiasmata”. These chiasmata result from the preliminary formation of crossovers (COs), a reciprocal exchange of large DNA fragments between homologues (Blary and Jenczewski, 2019). At least one CO (termed the “obligatory CO”) is formed between two homologous chromosomes in all species studied, but in general, no more than three COs per pair of homologues may occur (Girard et al., 2023). Due to the chiasmata, the pairs of homologues form structures called bivalents which are essential for their even separation and the successful progression of meiosis.

Meiotic COs are classified into two distinct types: Class I and Class II (reviewed in Girard et al., 2023). Class I COs rely on the ZMM protein complex (named as it according to *Saccharomyces cerevisiae*’s protein nomenclature: ZIP1/2/3, MSH4/5, and MER3). These COs are submitted to interference resulting in their non-random distribution along the chromosomes than what would be expected by chance. In contrast, Class II COs do not rely on the ZMM complex and are not subjected to interference. They are therefore distributed independently from one another and from class I COs.

Because COs participate in allelic shuffling over generations they have thus important consequences on genome evolution, on plant and animal breeding and on agronomical applications. However, because of the tight control of CO occurrence, only large blocks are exchanged and some regions like peri-centromeres are often deprived of CO events preventing gene admixture in these areas. This variation of CO rates in different regions of the genome results in a major bottleneck in plant breeding. There is therefore a need to improve recombination rate to create more easily and more rapidly new original combinations of genes/alleles that will be able to face the challenge of producing more and in a sustainable manner (Feuillet et al., 2008).

Several proteins have been shown to affect CO rate in the plant model species *Arabidopsis thaliana* (reviewed in Séguéla-Arnaud et al., 2015). Interestingly, some of these proteins – namely FANCM, FIGL1, and RECQ4 – have been the focus of intense research, particularly in the field of crop genetics like pea (*Pisum sativum*), rice (*Oryza sativa*), and tomato (*Solanum lycopersicum*) leading to contrasted results depending on the species (Mieulet et al., 2018). *AtFancM* was the first to be discovered (Crismani and Mercier, 2012; Crismani et al., 2012) but even if its mutation leads to almost a three-fold increase of recombination rate in pure lines, the effect remains very limited in Arabidopsis hybrids (Fernandes et al., 2018). Mutation of *FancM* does not change the fertility in pea and rice and leads to a two-fold increase of recombination in hybrids (Mieulet et al., 2018). A similar analysis was conducted in durum (*Triticum turgidum* ssp. *durum*) and bread (*T. aestivum* L.) wheats (Desjardins et al., 2022) where mutation of *fancM* resulted in a significant loss of fertility in both species (respectively 36% and 15%) and a mitigated effect on recombination rate (>10% of CO increase in 11 intervals; >10% of CO decrease in five intervals; no difference in four intervals).

Mutation of *Atfigl1* has a limited effect on recombination in Arabidopsis hybrids (25% increase; (Fernandes et al., 2018)) but a similar mutation in pea, rice and tomato induces a complete sterility in these three species (Zhang et al., 2017; Mieulet et al., 2018). However, it was demonstrated that the simultaneous mutation of *Atrecq4A*, *Atrecq4B* and *Atfigl1* in Arabidopsis resulted in a 7.8-fold increase of genome wide CO rate (Fernandes et al., 2018). Similarly, mutation of *recQ4* alone in pea, rice and tomato hybrids results in 4.7, 3.2 and 2.7 CO increase respectively albeit a loss fertility in pea (4-times less seeds than the wild-type; (Mieulet et al., 2018)). The effect of the mutation of *SlrecQ4* in tomato was confirmed with a 1.5-fold increase in the number of COs (Maagd et al., 2020). These findings confirm that RECQ4 protein is the most potent anti-CO factor actually identified and demonstrate its crucial role in regulating CO frequency.

RECQ4 (the Slow Growth Suppressor 1 (Sgs1)/Bloom (BLM) syndrome protein homologs) is a helicase that plays a critical role in restraining COs by preventing DNA intermediates (Higgins and Green, 2008; Higgins et al., 2011), such as Holliday junctions, from undergoing migration and ultimate dissolution before the occurrence of deleterious CO events (Wu et al., 2005). RECQ4 helicases are present in both prokaryotes and eukaryotes and exhibit various numbers of copies, ranging from one in *Escherichia coli* to eight in some plant species (Hartung et al., 2007). These helicases act upon various DNA structures, including D-loops, forked duplexes, or triple helices, by unwinding DNA in a 3’ to 5’ direction (Hartung and Puchta, 2006; Vindigni and Hickson, 2009; Kaiser et al., 2017). AtRECQ4A protein seems preferentially associated at the telomeres and could be involved in the removal of spurious recombination-dependent telomeric associations (Higgins et al., 2011). Combination of *AtrecQ4A/B* and *Atfancm* mutations shows that these two helicases affect class II COs without affecting the number of class I COs but that they act independently, probably by processing different substrates (Séguéla-Arnaud et al., 2015). All these results suggest that manipulating *RecQ4* gene in crops could be of main interest in breeding programmes.

Among the crops that need to be improved, bread wheat (*T. aestivum* L.) is one of the most important since it represents 20% of the calories consumed (Feuillet et al., 2008) and the second most widely cultivated crop in the world (Intercéréales, 2021). Bread wheat is an allo-polyploid species derived from two natural successive interspecific hybridizations involving three related diploid species that diverged 5-7 Million years ago (Marcussen et al., 2014). The first hybridization event aroused between *T. urartu* (AA genome), and a species related to *Aegilops speltoides* (S genome parented to the B genome of wheat) about 0.8 Mya. This resulted in the creation of *T. turgidum*, the ancestor of the actual pasta wheat (ssp. *durum*; AABB). This tetraploid species hybridized with *Ae. tauschii* (DD) < 0.4 Mya to give rise to the actual hexaploid bread wheat (Marcussen et al., 2014). In bread wheat, 80% of COs occurs in 20% of the genome, with at least 50% of wheat genes lying in recombination poor regions (Saintenac et al., 2009; Choulet et al., 2014; Alaux et al., 2018; Danguy des Déserts et al., 2021). Improving recombination rate in this species is therefore of main interest and looking deeper in the effect of the mutation of *RecQ4* could be a track to follow.

The objective of our study was therefore to investigate the effects of *TaRecQ4* mutation in bread wheat. As an allo-hexaploid species, *TaRecQ4* is present in six copies (one copy on each homologue of the A, B & D genomes) and locates on the long arm of chromosomes from homoeologous group 2. We generated single (two copies from one homoeologous pair knocked out; named hereafter aaBBDD, AAbbDD, AABBdd), double (aabbDD, aaBBdd, AAbbdd) and triple (aabbdd) *Tarecq4* mutants in the variety Renan as well as heterozygous mutants for one copy (Aabbdd, aaBbdd, aabbDd) to study how the complete mutation of *TaRecQ4* affects the morphology and fertility of the plants. We compared the sequences of the three homoeologous copies in a wide range of genotypes to estimate their functionality. These findings highlight the critical role of *TaRecQ4* in the maintenance of chromosome integrity and meiotic fidelity in wheat and provide a basis for further investigations into the mechanisms underlying these processes.

## Material and methods

### Plant material and greenhouse conditions

To investigate the impact of the mutation of *TaRecQ4*, we screened (Q-PCR) an in-house collection of 4500 irradiation lines (150 γ-rays) from Renan variety to isolate single-copy mutants for each of the three homoeologous copies. We then manually and reciprocally crossed each mutant (**Supplementary Figure 1**) to generate double mutants that were further also reciprocally crossed to generate hybrids for the three copies (AaBbDd). These triple hybrids were then self-pollinated to generate a collection of lines from which, using Q-PCR, we isolated 57 plants with either no (wild-type; AABBDD), one (aaBBDD, AAbbDD, AABBdd), two (aabbDD, aaBBdd, AAbbdd) or three (aabbdd) mutated copies, along with plants exhibiting heterozygosity for one copy and homozygosity for the mutation for the other two copies (Aabbdd, aaBbdd, aabbDd). Three different individuals (replicates) for each combination were randomly selected for further analyses. Seeds were sown in potting soil and grown until the three-leaf stage. They were then transferred in a vernalization chamber at 6°C ± 1°C during two months with an 8h/16 h day/night alternance. The plants were then potted in a 4-litre pot containing Nutricote (Fertil, a commercial progressive release fertilizer) and placed in a greenhouse under a 16h/8h and 23°C/18°C day/night photoperiod and temperature. At the time of transplanting, 100 mg of fresh leaves were taken for DNA extraction and molecular analyses.

### Identification of *TaRecQ4* copies

DNA and protein sequences of the three homoeologous copies of *TaRecQ4* in the cultivar Renan were recovered through a BLAST-n search using Arabidopsis DNA sequence of *AtRecQ4A* (AT1G10930) as query. This resulted in the identification of the three homoeologous sequences: *TraesRN2A0100736900*, *TraesRN2B0100862400*, and *TraesRN2D0100744400* that will be further named as *TaRecQ4-A*, *TaRecQ4-B* and *TaRecQ4-D* respectively. In addition, sequences from 15 other varieties (Chinese Spring, ArinaLrFor, Cadenza, Claire, Jagger, Julius, Lancer, Landmark, Mace, Norin61, Paragon, Robigus, Stanley, Sy-Mattis, Weebill) and two related species (*T. dicoccoides*, AABB; *T. urartu*, AA) were retrieved from the 10+ genomes sequences available on Ensembl (https://plants.ensembl.org/index.html; (Walkowiak et al., 2020)) using the same approach. Sequences were aligned using the Clustal Omega method in UGENE (Okonechnikov et al., 2012). From these sequences, pairs of primers that were specific to each homoeologous copy were designed (**Supplementary Table 1**) for further screening of the irradiated population.

### Anthers and meiocytes isolation

To analyse the meiotic behaviour of the *TaRecQ4* mutants, immature inflorescences were collected in the morning and placed immediately on ice. The ears were carefully removed from the sheath, and the three anthers of each flower were extracted. One of the anthers was stained with Acetocarmine (10 g L^-1^ Carmin, 45% acetic acid) and observed under a microscope to identify the meiotic stage. The remaining two synchronized anthers were fixed in Carnoy solution (EtOH 100% - glacial acetic acid; v/v 3:1) for 48 hours, followed by storage in EtOH 70% at 4°C. To prepare the meiotic atlas, one anther from each stage was placed on a poly-L-lysine-coated slide with a drop of acetocarmine and opened using two roll pins under binocular to expose the meiocytes. A drop of acetic acid 45% was applied to remove acetocarmine, and the slides were immersed in liquid nitrogen, air-dried briefly, and mounted with Vectashield-DAPI (Eurobio-Ingen). Images were captured using an Axio Observer Z1 fluorescence microscope with Zen software (Carl Zeiss microscopy). The chromosome spreads were prepared from anthers fixed at metaphase I stage by treating with 45% acetic acid and heating on a hotplate at 90°C for 2 to 3 seconds until the chromosomes were sufficiently separated (Jahier and Tanguy, 1992; Chen et al., 1995; Chen et al., 2001). For meiotic behaviour studies, a minimum of one hundred cells per genotype were imaged under a brightfield light Zeiss Axio Observer Z1 microscope, and the mean numbers of chiasmata and pairing types were calculated.

### Immunofluorescence and confocal microscopy

To investigate the establishment of synaptonemal complex at prophase I, we used immunofluorescent antibodies raised against ASY1 and ZYP1 proteins. Fresh immature anthers were extracted and prepared as previously described in Benyahya et al. (2020). Briefly, 50 µL of primary-antibody solutions [1:200 dilution of anti-AtASY1 (rabbit, Phyto-AB USA) and 1:150 dilution of anti-TaZYP1 (guinea pig) supplied by A.C. Martin, John Innes Centre, UK] were deposited per slide and covered with a piece of parafilm. The slides were then placed in a humid chamber at 4°C for 48 hours. After three washes of 5 min. with PBS 1X, the secondary-antibody solutions [1:400 dilution of anti-rabbit Alexa Fluor 488 and 1:300 dilution of Alexa Fluor 647 (Fisher Scientific), both in BSA 5%] were applied for 1 hour in the dark for the detection. The slides were washed three times with PBS 1X and then mounted in Vectashield-DAPI. Fluorescent images of meiocytes were acquired using a confocal LSM 800 microscope (Carl Zeiss) and analysed with Zen2 image analysis software parameters, as previously described in Benyahya et al. (2020).

### Pollen viability and fertility

Fresh pollen from one anther was collected and stained using Alexander reagent for 3 minutes as described in Jahier and Tanguy (1992). The experiment was conducted with two biological replicates per genotype, and four technical replicates per biological replicate. Slides were observed under brightfield light using a Zeiss Axio Observer Z1 microscope and full field images were acquired using the Tiles module on Zen2 image analysis software. The number of viable pollen grains (magenta staining) and non-viable pollen (blue staining) was counted using Image J software, and the percentage of viable pollen, means and standard deviations were calculated.

For the fertility study, three plants per genotype were selected and 3-4 master spikes per plant were bagged for self-pollination. The number of mature grains per spike was counted and the means and standard deviations per genotype were calculated.

### Statistics

The Windows Excel software was used to perform statistical inference tests. Normality of the values was assessed using the normality test of Shapiro and Wilk with default values. If the values were found to follow a normal distribution, a two-sample student’s T-test was used with the default settings (α = 0.05). Alternatively, if the values did not follow a normal distribution, we applied a non-parametric Kruskal-Wallis test with an α error of 0.01. For fertility, T-test was used to compare the means and determine if they were significantly different with a probability threshold of p < 0.05.

### Protein structure and sequences analysis

To obtain the three-dimensional (3D) structure of the proteins, protein sequences were uploaded onto the Phyre2 server (Structural Bioinformatics Group Imperial College London et al., 2011; Kelley et al., 2015) for basic analysis. The resulting 3D structures were found to be similar to RECQ4 Human protein and were downloaded in PDB format. Analysis of the structures was performed using ChimeraX desktop software (Goddard et al., 2018; Pettersen et al., 2021). Motif search in the protein was carried out using the Motif Search tool provided by GenomeNet (Kanehisa, 1997; GenomeNet, 2023), and the identified motifs were highlighted on the 3D protein using Chimera. The alignment of sequences was performed using clustal omega (Sievers et al., 2011).

### Molecular analysis of the mutants

Each mutant was genotyped using an in-house 35K Affymetrix Axiom genotyping SNP array (Rimbert et al., 2018). The analysis uses the allele calls and signal intensities, which were generated using the genotyping pipeline as previously described (Balfourier et al., 2019). For each accession and SNP, a one-dimensional measure of signal intensity at a given marker was calculated as a log r value. This log r value was then normalized using the wild-type parent of the population as a reference (Renan). A negative value indicated a SNP for which the accession had a signal intensity weaker than that of the reference, with the decrease in signal following a log2 scale. We then applied a segmentation algorithm to these values, seeking for breakpoints in the normalized signal intensity for a given chromosome and accession. The R package changepoint (Killick and Eckley, 2014) was used to conduct the segmentation step, searching for changes in mean using the default parameters for the binary segmentation algorithm. We then detected candidate segments containing stretches of low signal as described in De Oliveira et al. (2020), which suggested the presence of chromosomal regions potentially deleted by γ-rays. To further investigate these candidate segments, we used allele calls to verify if there were many Off Target Variants (OTVs) within the detected boundaries, which would further confirm that the segment of interest is deleted. However, this approach was limited in detecting segments due to low SNP density on certain chromosomes and short sizes of variations. Therefore, outlier detection was added to the analysis, using a linear regression of normalized signal intensity versus SNP position along the chromosome. An outlier Test was then conducted, using the R package *car* (Fox and Weisberg, 2019). When no changepoint was detected in the normalized intensity signal, a chromosomal region containing a high density of OTVs, outliers and weak signal SNPs was considered as a candidate region for deletion.

### Identification of deleted genes

To identify genes that were deleted simultaneously with *TaRecQ4*, we established a list of genes between the deletion borders using the gene annotation from the reference-quality assembled genome sequence of Renan (Aury et al., 2022) (https://www.ebi.ac.uk/ena/browser/view/GCA_937894285). The coding sequences (CDS) of these genes were translated into protein sequences and subjected to an all-against-all BLAST alignment using OrthoFinder v2.5.4 (Emms and Kelly, 2019), with the parameter *y* enabling splitting paralogous clades. The resulting orthologous groups allowed to determine which homoeologous genes were deleted in only one, two or all three homoeologous chromosomes. To evaluate the biological consequences of gene deletions concomitant with *TaRecQ4*, we retrieved the functional annotations of the corresponding genes in Chinese Spring (The International Wheat Genome Sequencing Consortium (IWGSC) et al., 2018), and used the integrated knowledge network KnetMiner (Hassani-Pak et al., 2021) to investigate potential impact on meiosis or COs regulation from co-deleted genes.

## Results

### Comparative analysis of wheat *TaRecQ4* copies

As expected, we identified three homoeologous copies of *TaRecQ4* in the wheat genome: *TaRecQ4-2A*, *TaRecQ4-2B* and *TaRecQ4-2D*. These three copies were identical in structure (25 exons; **Table 1**; **Supplementary Table 2**) as well as in CDS and protein lengths. They are similar to AtRecQ4A/B with their 25 exons, differing however in size of CDS (**Supplementary Table 2**).

**Table 1:** Comparative analysis of wheat Renan *TaRecQ4* genes and proteins of each sub-genome.

Renan	Genomic length (bp)	cDNA length (bp)	Number of exons	CDS length (bp)	Protein length (aa)
*TaRecQ4-2A*	9342	4381	25	3606	1201
*TaRecQ4-2B*	8994	4259	25	3606	1201
*TaRecQ4-2D*	9010	4102	25	3606	1201

Comparative analysis between the three A, B, and D CDS copies indicated 98.2% (AB), 98.2% (AD), and 98.6% (BD) nucleotide identity and 98% (AB), 98% (AD), and 98% (BD) of protein identity. These results suggest that the three homoeologous copies are highly similar and could complement each other if one or two is(are) mutated.

### Phenotypic and genotyping analyses

**Figure 1:**
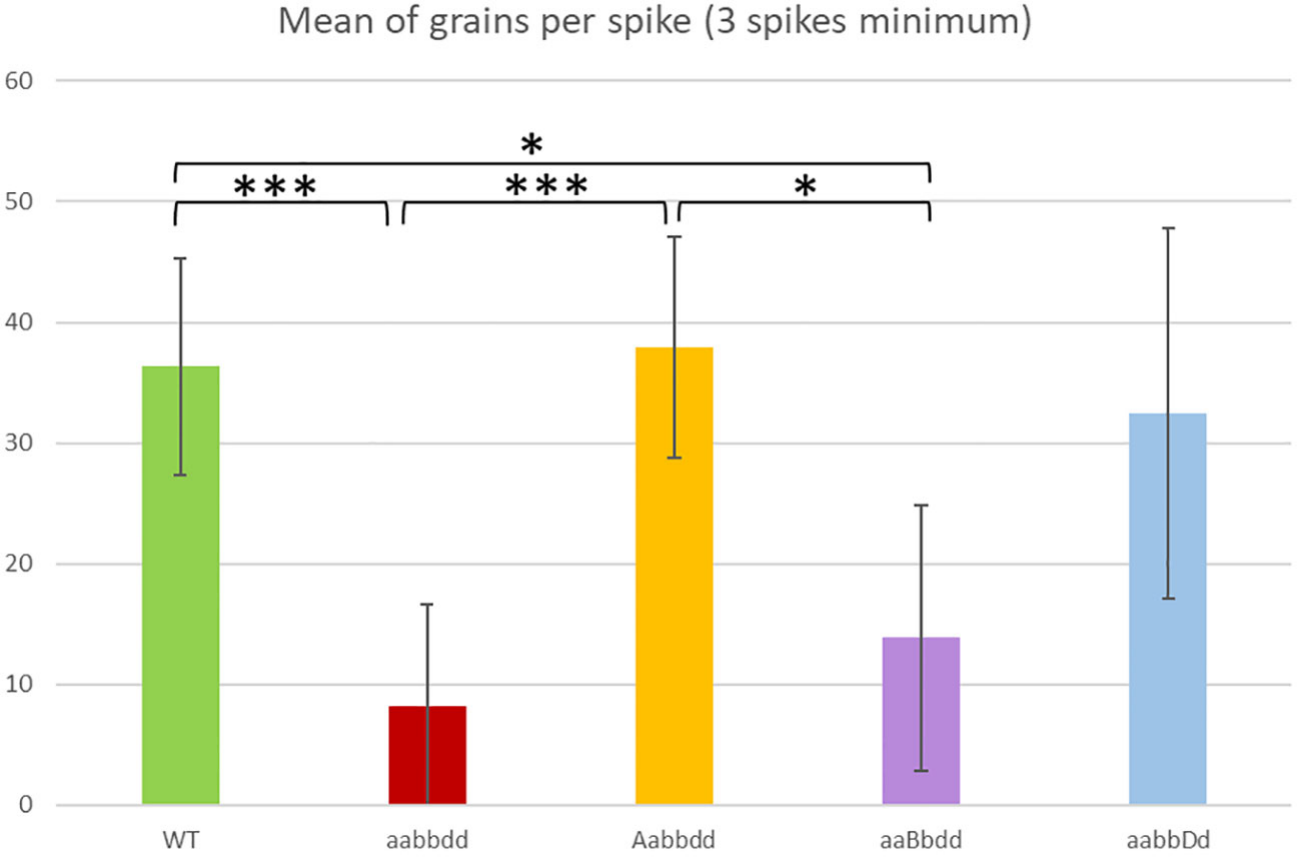
Mean number of grains per spike for the variety Renan wild-type (WT), for the individuals with one heterozygous copy (Aabbdd, aaBbdd, aabbDd) and for the triple mutants (aabbdd). A minimum of three spikes per individual was recorded. * = p < 0.05, *** = p < 0.001. The values of T-Test are given in **Supplementary Table 3**.

We investigated the phenotype of triple mutants (TM plants, aabbdd) for *TaRecQ4* compared to wild type plants (WT plants, AABBDD), which had the same genetic background but no mutation on *TaRecQ4*. Phenotypic observation did not reveal any visible difference concerning plant morphology between TM and WT (**Supplementary Figure 2**). No phenotypic difference was observed in heterozygous for one copy and mutant for the two others (Aabbdd, aaBbdd or aabbDd plants) compared to WT either. We recorded the number of seeds per individual (**Figure 1**). The plants that were heterozygous for the A or D copies (Aabbdd (Htz-A) or aabbDd

(Htz-D)) were not affected in their fertility compared to WT (p > 0.5). On the contrary, we found a significant reduction in seed sets in TM plants compared to WT plants (8 seeds/spike vs 36 seeds/spike; p < 0.001). Interestingly, the seed set of plants that were heterozygous for the B copy but mutated for the A and D copies (Htz-B, aaBbdd) was also significantly reduced (mean 15 seeds/spike; p < 0.05) compared to WT plants and to Htz-A (38 seeds/spike) but the difference was not significant with Htz-D (32 seeds ± 15/spike, **Supplementary Table 3**).

To confirm this result, we checked the viability of the pollen in the different mutants. We found a significant reduction in pollen viability in TM plants compared to WT plants (p-value < 0.001) but a lower significance with Htz-A (p-value < 0.01) and Htz-D plants (p-value < 0.05) (**Supplementary Figure 3**). No significant reduction was observed in Htz-B plants versus WT or TM plants despite B copy plants exhibiting partial sterility. Since *RecQ4* has never been described as leading to severe sterility (Fernandes et al., 2018; Mieulet et al., 2018) and because irradiation is known to create deletions in the genome (reviewed in Aklilu, 2021), we genotyped each single mutant with a high-density SNP array (AXIOM™, see Experimental procedure section) to estimate the size of each deletion. Our results showed that the mutations on each chromosome were highly targeted and specific (**Supplementary Figure 4**). Deletion size was estimated to 11.6, 16.4 and 18.9 Mb for *TaRecQ4-A*, -B and -D respectively. This represented 95, 84 and 163 genes respectively among which 46 were common between the three deletions. Among these 46 genes deleted together with *TaRecQ4*, 21 were annotated: *ASK1*, *BAT1*, CINV1, *CR4*, *ER2*, *GLTP2*, *NAC104*, *NAM-1*, *NPK1*, *OGR1*, *PDIL1-1*, *PDIL5-2*, *PME64*, *PPD2*, *RAM1*, *RGA2*, *SBT3*, *SKIP32*, *TCHQD*, *UVR8* and *ZHD5*. Only *ASK1* (named *TaSKP1* in wheat) has been previously identified as essential for proper chromosome segregation (Zhao et al., 2003; Li et al., 2006). However, it has been previously shown that every chromosome in wheat contains at least one copy of *TaSKP1* (Beji et al., 2019). This suggests that the loss of fertility is rather due to the deletion of *TaRECQ4* or another unknown gene, but not to that of *TaSKP1*.

All our results suggest that the complete mutation of *TaRecQ4* (all six copies knocked out) induces plant sterility and loss of pollen viability. However, heterozygous A and D mutants (Aabbdd and aabbDd respectively) exhibit a normal fertility suggesting that one copy among six is enough to ensure pollen viability and fertility and that there is no dosage-effect for *TaRecQ4*. Interestingly, the *TaRecQ4-B* copy is probably very poorly or even not functional in Renan.

### Meiotic behaviour of mutants

**Figure 2:**
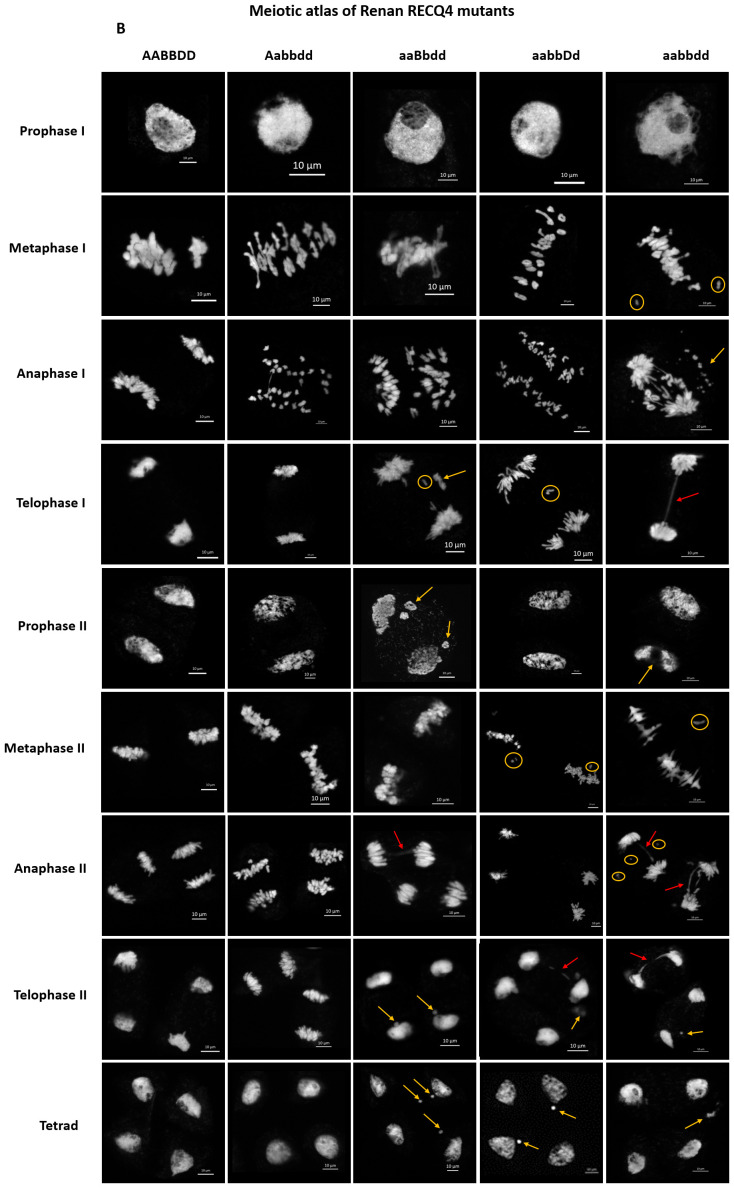
Meiotic atlas from prophase I to tetrad of Renan and mutants for *TaRecQ4*: AABBDD: WT; Aabbdd: Htz-A; aaBbdd: Htz-B; aabbDd: Htz-D; aabbdd: TM. Abnormalities are indicated as follows: orange circle: univalent; orange arrow: chromosome fragmentation; red arrow: bridge. Scale bar: 10 µm.

We generated a meiotic atlas for Renan *TaRecQ4* mutants versus WT to detail and to compare their meiotic behaviour (**Figure 2**). We found no difference between WT and Htz-A plants confirming that *TaRecQ4-A* copy is fully functional, and that one copy of *TaRecQ4-A* is enough to perform meiosis. For the other mutants at prophase I, we could see fragmentation of the chromosomes at pachytene stage for TM and Htz-B and at diplotene and diakinesis stages for Htz-B and Htz-D (**Supplementary Figure 5**). During the first meiotic division, the TM plants exhibited the most defaults with the presence of univalents at metaphase I, fragmentation, and presence of bridges at anaphase and telophase I. These abnormalities were confirmed for TM during the second meiotic phase. We observed unaligned chromosomes at metaphase II and presence of univalents as well as bridges at anaphase II with fragmentation starting from prophase II up to tetrad. Htz-B exhibited the most cells with univalents at metaphase I (up to 7.6%, **Supplementary Table 4**) followed by Htz-D which showed the presence of univalents at metaphase I (5%), telophase I and metaphase II stages. During anaphase II, bridges were observed for both TM and Htz-B and fragmentation was visible from telophase I to tetrad for Htz-B.

Based on that, we can conclude that, since Htz-A had similar meiotic behaviour as WT and Htz-D presented only limited meiotic defaults in a few cells (a few fragmentations at the end of prophase I as well as a few univalents) and a normal fertility, both *TaRecQ4-A* and *TaRecQ4-D* copies are likely functional in Renan. TM and Htz-B are those for which meiosis is the most disrupted with early DNA fragmentation abnormalities starting from prophase l (pachytene and up to the tetrad) and frequent occurrence of univalents and anaphase’s bridges leading to severe sterility in both individuals. Therefore, *TaRecQ4-B* is probably fully knocked out in Renan.

### Effect of *TaRecQ4* mutation on pairing forms and chiasma frequency

**Figure 3:**
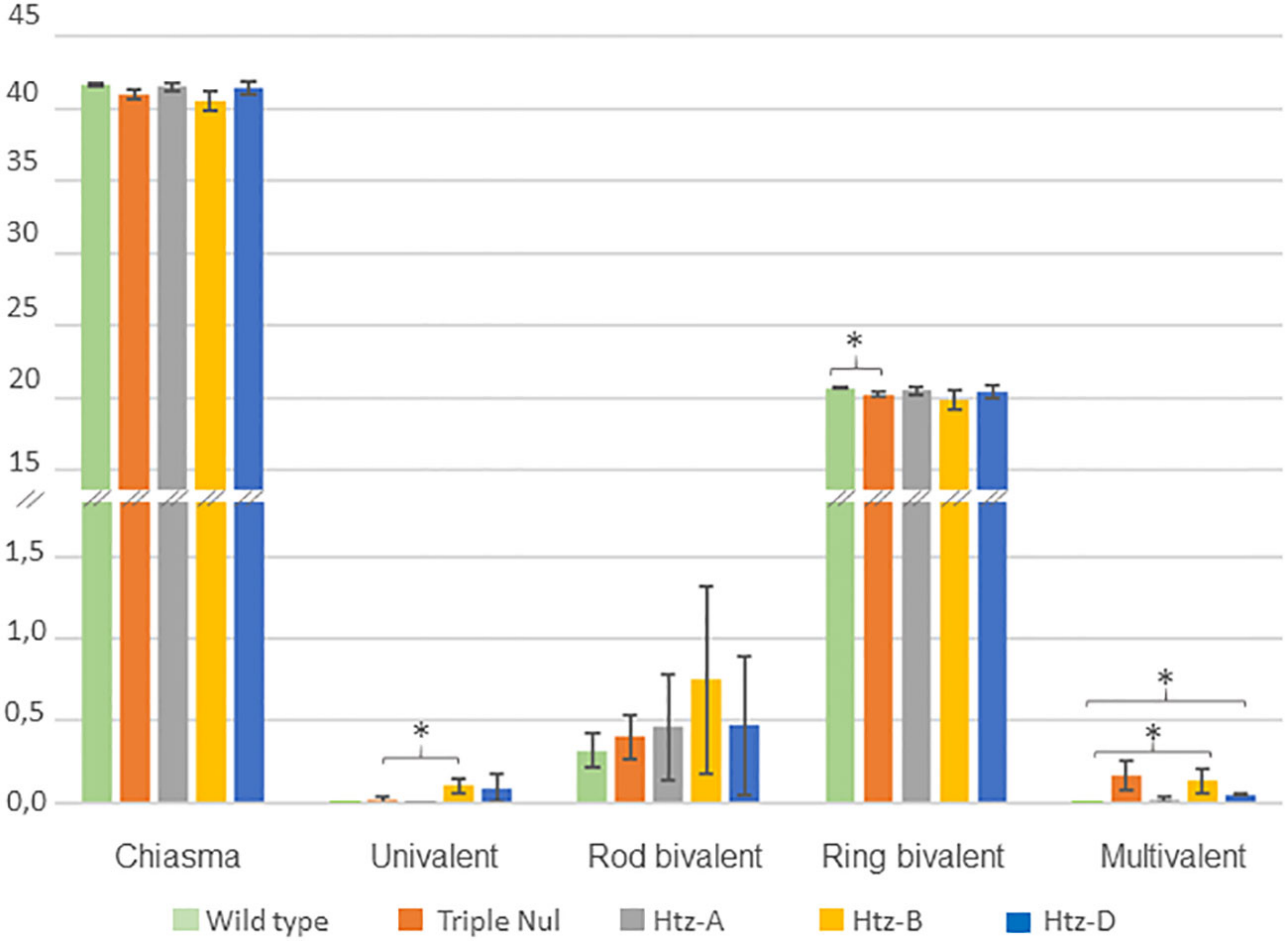
Meiotic behaviour of WT and *TaRecQ4* mutants (Htz-A, Htz-B, Htz-D and TM). The vertical axis represents the numbers and forms of chiasmata. Means and standard deviations were calculated for chiasma, ring bivalent, univalent, rod bivalent and multivalent numbers. One star means that T. Test was significant at p-value < 0.05.

Since *RecQ4* mutation is known to affect CO frequency (Fernandes et al., 2018), we investigated this parameter in the WT and TM individuals. To achieve this, we applied chiasma counting since chiasmata are the visible representation of COs and are known to be a reliable indicator of recombination intensity (**Figure 3**).

As expected, the WT Renan exhibited 41.68 ± 0.103 chiasmata with a large majority of ring bivalents (20.20 ± 0.136), a few rod bivalents (0.32 ± 0.103) and neither univalents nor multivalents (**Supplementary Table 5**). This corresponds to what is usually observed for wheat wild-type varieties (Benyahya et al., 2020). WT presented the lowest percentage of rod bivalents compared to *TaRecQ4* mutants. Interestingly, the mean number of chiasmata observed at metaphase I in TM plants was not significantly different from that observed in wild-type (WT) plants (41.03 and 41.68 respectively, p-value > 0.05). However, the number of ring bivalents of TM was significantly different from WT, p-value=0.032 (**Supplementary Table 6**; **Figure 3**).

Since the most frequent form for all individuals was ring bivalents, we counted the number of cells (range 100-190 cells; **Supplementary Table 4**) with one to three alternative shapes (univalents, rod bivalents, trivalents, ring and rod quadrivalents, complex shapes). Htz-A and Htz-D showed a similar percentage of cells with rod bivalents (40.9-39% respectively) and Htz-A had the lowest percentage of multivalents (2%). Htz-D showed the same percentage of univalents and multivalents (5%). The number of multivalents was significantly different between Htz-D and WT (**Supplementary Table 6**; **Figure 3**). Htz-D showed only trivalents (with 2 chiasmata) and ring quadrivalents (with 4 chiasmata) but no more complex forms were found.

Htz-B was the heterozygous copy that had the most cells with univalents (7.6%) and rod bivalents (49.3%). Htz-B had almost five times more univalents than TM (significant T-test, p-value=0.04) and a similar percentage of multivalents (10.4% and 11.6% respectively) with a vast majority of complex forms that was significantly different from that of WT, Htz-D and Htz-A (**Supplementary Table 6**; **Figure 3**).

### Meiotic behaviour using immunofluorescence

**Figure 4:**
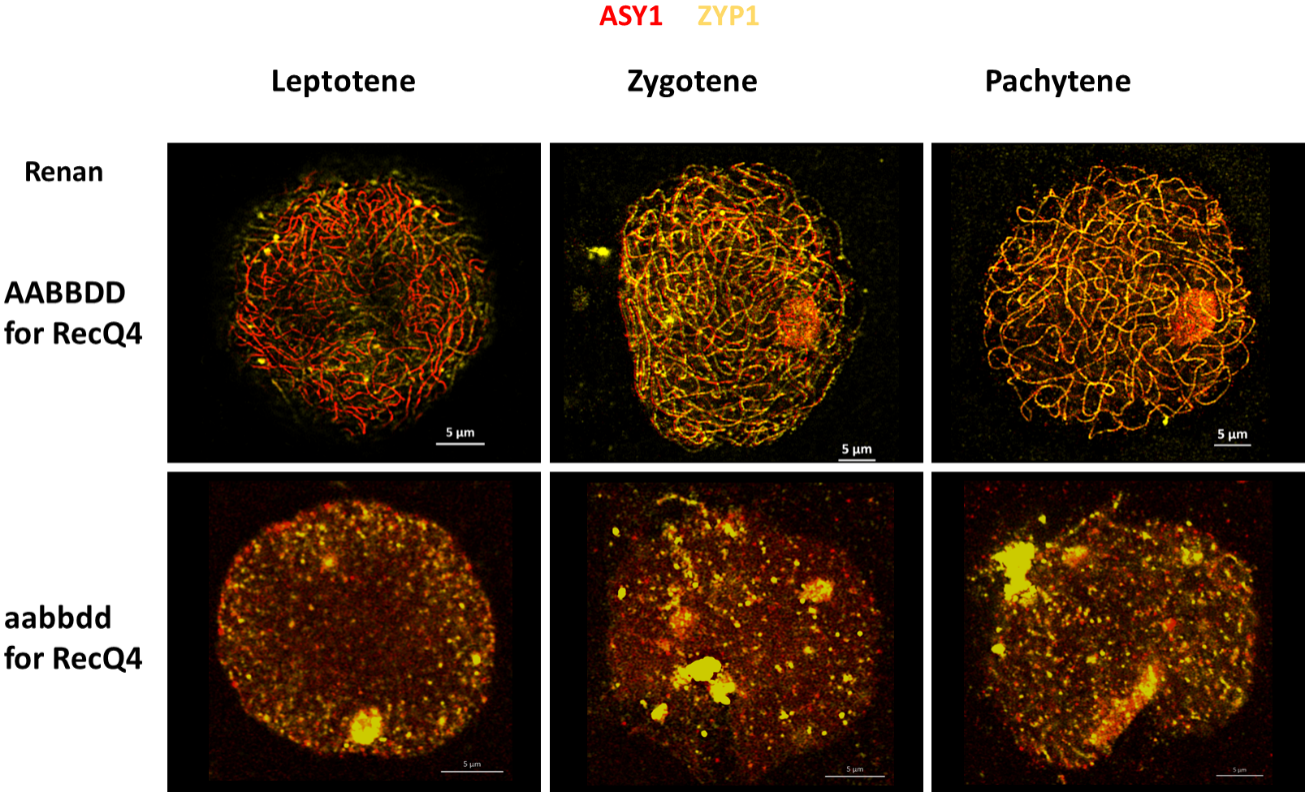
Meiotic behaviour of wheat chromosomes during Prophase I in Renan WT (AABBDD) and Triple Mutant (aabbdd) for the *TaRecQ4* gene. ASY1 is labelled in red and ZYP1 in yellow. Scale bar 5 µm.

We followed accurate synapsis of homologous chromosomes during Prophase I of WT and TM with antibodies raised against ZYP1 (yellow) and ASY1 (red) to examine potential differences in chromosome fragmentation between all individuals (**Figure 4**). In the wild type (Renan AABBDD), synapsis between homologues starts at the telomeres in the early steps of leptotene and progresses during zygotene. Synapsis is fully completed at the end of pachytene with a co-localization of ASY1 and ZYP1 proteins. The TM plants exhibited distinct chromosomal fragmentation from leptotene to pachytene, represented by dots, when compared to WT where full synapsis was observed. These dots indicate DNA fragmentation prior to compaction of the chromosomes on the metaphase plate and an impossibility to complete synapsis. These observations suggest that the TM plants may exhibit significant alterations in the structure of chromosomes during meiosis, as shown previously with the atlas. Based on our results, *TaRecQ4* should affect DNA reparation at early meiotic phases.

### Expression and structural analyses of the *TaRECQ4* proteins

The expression of the three *TaRecQ4* copies was investigated using available RNA sequencing data for Chinese Spring anthers during meiosis (Lloyd et al., 2014; **Supplementary Figure 6**). All three copies were expressed at the same level suggesting that the loss of efficiency of *TaRecQ4-B* is due to a variation in the CDS sequence that may result in a variation in amino acid (aa) sequence and further in its 3-dimensional conformation.

We identified a A>G mutation at position 1615 bp in the CDS of *TaRecQ4-B* compared to *TaRecQ4-A* and *TaRecQ4-D* sequences, leading to a Threonine>Alanine change at aa position 539 (T539A). This change occurs in the helicase ATP bind/DEAD/ResIII domain which unwinds nucleic acids and is crucial in rice (Mieulet et al., 2018). Replacing Threonine, a big neutral amino acid, by Alanine, a very little hydrophobic amino acid, may alter the activity of this domain and impair the protein’s ability to unwind nucleic acids.

We compared the DNA and protein sequences of *TaRecQ4-B* among all the wheat genome sequences available on EnsemblPlants database (Walkowiak et al., 2020)⁠. We found that 15 of the 16 sequences available for *TaRecQ4-B* had this A>G mutation, resulting in the T539A substitution in the DEAD domain (**Supplementary Figure 7**; **Supplementary Table 7**). Only the LongReach Lancer accession does not present this mutation, which can be explained by the introgression of a DNA segment coming from *Triticum timopheevii* on chromosome 2B (Walkowiak et al., 2020); matrix of identity **Supplementary Table 7**). As expected, the *AesRecQ4* sequence of *Aegilops speltoïdes* (Yang et al., 2023), the most proximal donor of the B genome of wheat, is not mutated confirming its functionality in this species.

Similarly, we investigated sequences derived from exome captures of 811 wheat varieties (URGI JBrowse database, https://urgi.versailles.inra.fr/jbrowseiwgsc/gmod_jbrowse/)) and found that the A>G mutation on *TaRecQ4-B* is present in 97.4% of the samples, indicating a high frequency of this mutation among wheat varieties. Our findings suggest that the observed differences in the activity of TaRECQ4 protein are due to differences in protein conformation caused by the single nucleotide modification, highlighting the importance of studying protein conformational changes in understanding its biological activity.

## Discussion

### Does *TaRecQ4* mutation induce sterility in wheat?

*RecQ4* has been recognized as the most potent gene to improve recombination rate in plants without affecting too much the fertility of the mutants (Mieulet et al., 2018). Here, we used irradiation mutants deleted for the three homoeologous copies of *TaRecQ4* to study the effect of its deletion on recombination rate in bread wheat. Deletion of this gene did not affect plant development but significantly reduced by 3-4 folds the number of seeds produced per spike. Similar results were observed for *FIGL1* in pea and tomato (Mieulet et al., 2018) while mutation of this gene does not affect fertility in Arabidopsis. The same holds true for *TaFancM* for which mutation in wheat (bread and durum) induces a decrease in seed number in both species (Desjardins et al., 2022). Our results confirm that data obtained in a model species are not always transferable to crops and detailed studies must be conducted in the species of interest.

We used irradiation mutants developed in cultivar Renan. Even though the overlapping of deletions was restricted to approximatively 15.6 Mb (respectively 11.6, 16.4 and 18.9 Mb for sub-genomes A, B and D), this included 46 high-confidence genes. Among these latter, only 21 were annotated but based on their function and on data from literature, the deletion of none of them could explain the loss of fertility. Only *TaSKP1*, which is involved in chromosome segregation, could affect meiotic behaviour (Beji et al., 2019). As this gene has a functional copy on each wheat chromosome, the deletion of the copies from homoeologous group 2 should not interfere with its role. However, we cannot exclude the fact that among the remaining 25 that are not annotated, the deletion of (at least) one could induce sterility.

To avoid the drawback of the deletions, it would be relevant to use other types of mutants such as those developed using Ethyl-Methyl Sulfonate (EMS). In wheat, EMS mainly induces single-base mutations (usually G/C to A/T changes) but deletions from a few bases to larger chromosome fragments may occur (Dong et al., 2009). For the variety Cadenza, 1203 EMS lines have been developed and their exome has been captured and sequenced (Krasileva et al., 2017). All the mutations have been collected and classified based on the predicted effect (stop codon, splicing variant, missense mutation; https://www.jic.ac.uk/). It would be relevant to use *TarecQ4* mutants from this collection to develop a similar material as we did with the Renan irradiation lines *i.e*., single, double and triple mutants. This would allow (1) to confirm or not the loss of fertility and (2) to make a cross with the triple mutant in Renan to derive a segregating population to measure genetically the variation of recombination rate.

A last approach to knock-out *TaRecQ4* would be to use transgenic technologies such as CRISPR/Cas system or Virus-Induced Gene Silencing (VIGS). These two techniques were proven useful in wheat for VIGS to reduce the expression of *TaFancM* (Desjardins et al., 2022) and for CRISPR/Cas to knock-out *TaSpo11-1* (Hyde et al., 2023).

### *TaRecQ4* mutation induces meiotic defaults in wheat

We observed meiotic defaults for the triple mutant lacking the three homoeologous copies of *TarecQ4*. This resulted in chromosome fragmentation during pachytene as well as at metaphase I stages, with the formation of anaphase bridges and presence of univalents. These fragmented chromosomes appeared to be poorly repaired, leading to problems in their separation resulting in non-viable gametes and fertility issues.

In Arabidopsis, *Atrecq4A* mutants are sensitive to methyl methane-sulfonate (MMS) and to Cisplatin, *i.e*., two chemicals that induce DNA damage (Hartung et al., 2007). More specifically, they are affected in repair events in which only one of the two DNA strands is blocked. RECQ4 is a member of the RTR complex (RECQ4-TOP3α-RMI1) that has been shown as a major non-crossover promoting factor during meiosis (reviewed in (Séguéla-Arnaud et al., 2017)). The N-terminus of the RECQ4A protein of Arabidopsis, which has more than 80% identity with TaRECQ4, is essential to repair DNA damages due to alkylation of DNA (Schröpfer et al., 2014). The presence of anaphasic bridges is similar to what we observed in *Tarecq4* mutants (**Figure 2**) as well as what can be observed at meiosis in Arabidopsis or maize RAD51 mutants (Su et al., 2017; Jing et al., 2019). In addition, *BLM*, the ortholog of *RecQ4* in Human, has been shown to be dispersed over the chromatin and associated with the synaptonemal complex during late pachytene and early diplotene (reviewed in Toledo et al., 2019), which is consistent with our observations of chromosome fragmentation during pachytene. This fragmentation was not repaired in our mutants, leading to problems in chromosome segregation during meiosis, as indicated also by the presence of anaphase’s bridges and univalents.

### Does *TaRecQ4* mutation increase chiasma number?

We did not observe a significant difference in chiasma number between WT and TM mutant for *TaRecQ4* (**Figure 3**). This could suggest that contrary to other species (Fernandes et al., 2018; Mieulet et al., 2018), mutation of *TaRecQ4* in wheat does not affect the rate of crossovers. However, because of the meiotic default that we saw in the triple mutant for *TaRecQ4*, we observed that 11.6% of the cells presented multivalents (more than two chromosomes paired) at metaphase I. In this case, chiasmata are more difficult to score and maybe their number is underestimated. To gain accuracy in chiasma numbering, it would be relevant to use specific antibodies designed against proteins marking class I (like HEI10; Desjardin et al., 2022) and class II (MUS81; Desjardins et al., 2020) crossovers.

At metaphase I, we expected the usual ring- or rod-bivalent shapes. However, the occurrence of numerous multivalents indicates that both homologues and homoeologous are associated at metaphase I in the triple *Tarecq4* mutants. This suggests that the mutation of *Tarecq4* in wheat may affect simultaneously homologous and homoeologous recombination. Similar results have also been observed in interspecific tomato hybrids (de Maagd et al., 2020). Only two genes known as involved in homoeologous recombination in wheat have been isolated to date: *Ph1* which has been identified as *TaZip4-B2*, a protein involved in the ZMM pathway for the type I crossovers (Rey et al., 2017); *Ph2* that was also isolated, and which corresponds to *TaMsh7-3D*, a protein from the mismatch repair (MMR) system (Serra et al., 2021). Recently, *TaAsy1*, which contributes to the formation of the axial element that tethers the two sister chromatids, was also shown as being involved in homoeologous recombination (Di Dio et al., 2023). Involvement of *TaRecQ4* in homoeologous recombination as well would suggest that there is a very sensitive balance between the two types of recombination (homologous and homoeologous) and that affecting one type results most of the time in affecting the second type. It would be interesting to see which genomes and/or chromosomes are involved in the multivalent formation using Genome *In Situ* Hybridization (GISH, Rey et al., 2021) and Fluorescent *In Situ* Hybridization (FISH, Harun et al., 2023) with chromosome-specific probes.

### TaRECQ4-A protein seems more efficient than TaRECQ4-B or -D

In triple mutant plants, which have a complete absence of TaRECQ4 protein, we observed more severe chromosome fragmentation during meiosis (and therefore more sterility) than in heterozygous plants, which have only one functional copy of the gene. Htz-A was as fertile as the WT (p-value=0.7) indicating that a single copy of TaRECQ4-A protein is sufficient for proper repair and chromosome segregation during meiosis. This also suggests that *TaRecQ4* does not act as a dosage-dependant manner in wheat. On the contrary, Htz-B was almost as sterile as the triple mutant (p<0.05) suggesting that *TaRecQ4-B* copy in Renan is not (or very poorly) functional. Interestingly, Htz-D was not significantly different from WT in terms of fertility (p-value=0.501) but it was not significantly different from Htz-B as well (p-value=0.441). This suggests that the efficiency of the three copies to produce grains could be classified, Htz-A being as efficient as WT, then Htz-D leading to less seeds and finally Htz-B being the less (or even not in Renan) efficient.

RNA-seq analysis revealed similar expressions of each *TaRecQ4* copy, suggesting that the activity reduction of the B copy was due to protein inactivation. It is known that homoeologous copies of genes in wheat may differ in functionality. For example, *TaGW2-D1* controls the size and weight of wheat grains, and its homoeologues vary in their functionality (Wang et al., 2018). We therefore investigated the DNA and protein sequences of the DEAD domain, which plays a crucial role in the helicase activity of RECQ4, as it provides the ATPase activity necessary for nucleic acid unwinding and remodelling (Séguéla-Arnaud et al., 2015). We revealed that TaRECQ4-A/TaRECQ4-D proteins have a Threonine (T) at position 539 in the DEAD domain while this aa is an Alanine (A) in TaRECQ4-B protein. This change could be responsible for either the inactivation or the reduction of activity of TaRECQ4B. In Drosophila, a single amino acid mutation of the DEAD domain in a RECQ protein led to the inactivation of the helicase activity required to unwind DNA (Hilbert et al., 2009). Mutation of the DEAD-like helicase domain (DEXDc domain) of *OsRECQ4* in rice resulted in protein inactivity, leading to a 3.2-fold increase in the total genetic map compared to wild type (Mieulet et al., 2018). Therefore, a modification in the DEAD domain of *TaRECQ4*, such as the T539A change that we observe in the *TaRECQ4-B* copy, may lead to a conformational change in the protein and the inactivation of its helicase activity. Our analysis of the putative 3D structure of the protein supported this hypothesis, revealing that even a small change in protein conformation due to a change in amino acid polarity can affect its functionality. Interestingly, analysis of the 10+ genomes sequences (Walkowiak et al., 2020) and the capture of 811 exome results (Pont et al., 2019) indicates that a majority of individuals (> 97%) do have the mutation in *TaRecQ4-B*. Those which do not have the mutation bear a DNA segment coming from *Triticum timopheevii* (tetraploid species; 2n = 4x = 28; genome AAGG) on chromosome 2B suggesting that this species restored the wild-type allele of *TaRecQ4-B*. This suggest also that mutation occurred: (1) either between ~4 and 0.8 Mya (Marcussen et al., 2014), after the divergence of *Aegilops speltoïdes* (since this species does not have the mutation) within the B lineage of wheat and before the tetraploidization; (2) after the hybridization giving rise to *T. turgidum* and that the mutation has been maintained in both tetraploid and after hexaploid wheats within the wheat lineage.

## Conclusion

We clearly showed that the mutation of *TaRecQ4* in wheat affects its meiotic behaviour and its fertility. However, we cannot state whether the mutation increases recombination rate or not. Mutation seems to have an impact on both homologous and homoeologous recombination. The *TaRecQ4-B* copy is inactive in the variety Renan but such mutation seems to be common among wheat diversity for this sub-genome. To further investigate this phenomenon, we should produce the triple mutant from EMS Cadenza ecotype of through CRISPR/Cas9 or VIGS approaches, in order to validate our results obtained with radiation. This will enable us to examine the effect of the inactivation of *TaRecQ4-B* copy on meiosis and on the formation of chiasmata in a more controlled and rigorous manner. This will also allow us to make the cross with the Renan triple mutant to measure accurately the variation of recombination rate by producing a segregating population and scoring variation of genetic distances. We should also validate the effect of the mutation on homoeologous recombination by crossing the triple mutants with *Aegilops variabilis* to observe potential occurrence of bivalents as it has been seen for *ph1* and *ph2* mutants (Rey et al., 2017; Serra et al., 2021). The results of this study will provide further insights into the role of TaRECQ4 protein in both homologous and homoeologous recombination and may contribute to the development of strategies to mitigate the defaults observed and improve the efficiency of recombination in wheat.

## Data availability statement

The datasets presented in this study can be found in online repositories. The names of the repository/repositories and accession number(s) can be found below: https://plants.ensembl.org/index.html, TraesRN2A0100736900.

## Author contributions

JB: Conceptualization, Data curation, Formal analysis, Investigation, Writing – original draft. IN: Data curation, Formal analysis, Investigation, Writing – review & editing. PL-Z: Data curation, Formal analysis, Investigation, Writing – review & editing. JK: Data curation, Formal analysis, Writing – review & editing. RDO: Data curation, Investigation, Writing – review & editing. FC: Data curation, Writing – review & editing. PS: Conceptualization, Funding acquisition, Project administration, Supervision, Validation, Writing – original draft.

## Supplementary material

Supplementary Table 1 Pairs of primers specific of each homoeologous copy of *TaRecQ4*.

Supplementary Table 2 Comparative analysis of wheat Renan *TaRecQ4* homoeologous genes and proteins for each sub-genome and of *Arabidopsis thaliana AtRecQ4A* and *AtRecQ4B*.

Supplementary Table 3 Values of Student Test obtained for comparative analysis of the number of grains between the variety Renan wild-type (WT), for the individuals with one heterozygous copy (Aabbdd, aaBbdd, aabbDd) and for the triple mutant (aabbdd). Significant values (p-value < 0,05) are indicated in green.

Supplementary Table 4 Percentage of cells with univalents, rod bivalents and multivalents for WT and each mutant for *TaReCQ4*.

Supplementary Table 5 Mean and standard deviation of chiasma number and pairing forms in Renan wild-type (WT) and TaRecQ4 mutants. Counting was performed on 120 cells for each genotype.

Supplementary Table 6 T. Test values obtained by comparative analysis of mutants between them and with WT for each pairing form category (rod bivalent, ring bivalent, multivalent) and no-pairing (univalent), p-value < 0.05. Boxes in green show the values for which the T test is significant. NA, not applicable.

Supplementary Table 7 Matrix of percentage of identity of *TaRecQ4* CDS for different wheat variety and for Arabidopsis (*AtRecQ4A*). The first line/column is for the variety we used in this article (Renan). Highlight in red are the 2 varieties without the T539A mutation. The last line/column is for Arabidopsis.

Supplementary Figure 1 Crossing scheme of wheat *TaRecQ4* mutants to obtain a triple mutant (TM) and heterozygous mutants on one copy of one sub-genome (Htz-A, Htz-B, Htz-D).

Supplementary Figure 2 Picture of Renan wild type on the left and the triple mutant for *TaRecQ4* on the right.

Supplementary Figure 3 Percentage of viable pollen for each category of mutants. T-Test was calculated and the significance was indicated by star(s): * p-value < 0.05; ** p-value p < 0.01; *** p-value < 0.001.

Supplementary Figure 4 Representation of OTVs and outliers, showing the deletion of the TaRECQ4 region on chromosomes 2A, 2B and 2D due to radiation.

Supplementary Figure 5 Prophase I atlas from leptotene to diakinesis for WT, Heterozygous Htz-A, Htz-B, Htz-D and TM mutants. The orange arrows underline the fragmentations of the nucleus in the mutants.

Supplementary Figure 6 level of expression of *TaRecQ4* homoeologous copies using RNASeq data from Lloyd et al., 2014. Lepto_1 & _2: leptotene stage; zygopachy_1 & _2: zygotene and pachytene stages; diplodia_1 & _2: diplotene and diakinesis stages; meta_1 & _2: metaphase I stage. Two replicates (_1 & _2) are done for each stage. *TaRecQ4* copies are colour-coded for the A- (yellow), B- (violet) or D (blue) genomes respectively.

Supplementary Figure 7 Structure and motifs of TaRECQ4 protein from Renan found with Motif finder.
