# Supplementary material for: TaRECQ4 contributes to maintain both homologous and homoeologous recombination during wheat meiosis
Source: Front Plant Sci. 2024 Jan 29;14:1342976. doi: 10.3389/fpls.2023.1342976 (PMC10859459; doi:10.3389/fpls.2023.1342976)
Supplement: Supplementary Table 1 — Pairs of primers specific of each homoeologous copy of TaRecQ4. [file Table_1.docx]

Table S.1: Pairs of primers specific of each homoeologous copy of *TaRecQ4*.

|  | Forward | Reverse |
| --- | --- | --- |
| RECQ4-2AL | AAGCACCTGTACAACGGC | AGCACCTACCGCCAGAAT |
| RECQ4-2BL | TGCTGTTCCAAATTATCCTTC | CACTGGCTTACACAATGAG |
| RECQ4-2DL | CCGCACTGAAAGTGGTG | AGCACCTACCGCCAGAAT |
