# Supplementary material for: TaRECQ4 contributes to maintain both homologous and homoeologous recombination during wheat meiosis
Source: Front Plant Sci. 2024 Jan 29;14:1342976. doi: 10.3389/fpls.2023.1342976 (PMC10859459; doi:10.3389/fpls.2023.1342976)
Supplement: Supplementary Table 2 — Comparative analysis of wheat Renan TaRecQ4 homoeologous genes and proteins for each sub-genome and of Arabidopsis thaliana AtRecQ4A and AtRecQ4B. [file Table_2.docx]

Table S.2: Comparative analysis of wheat Renan *TaRecQ4* homoeologous genes and proteins for each sub-genome and of *Arabidopsis thaliana* *AtRecQ4A* and *AtRecQ4B*.

| Renan | TaRecQ4-2A | TaRecQ4-2B | TaRecQ4-2D | AtRecQ4A | AtRecQ4B |
| --- | --- | --- | --- | --- | --- |
| Genomic length (bp) | 9342 | 8994 | 9010 | 7810 | 8031 |
| cDNA length (bp) | 4381 | 4259 | 4102 | 4029 | 3876 |
| Number of exons | 25 | 25 | 25 | 25 | 25 |
| CDS length (bp) | 3606 | 3606 | 3606 | 3567 | 3452 |
| Protein length (aa) | 1201 | 1201 | 1201 | 1188 | 1150 |
