# Supplementary material for: TaRECQ4 contributes to maintain both homologous and homoeologous recombination during wheat meiosis
Source: Front Plant Sci. 2024 Jan 29;14:1342976. doi: 10.3389/fpls.2023.1342976 (PMC10859459; doi:10.3389/fpls.2023.1342976)
Supplement: Supplementary Table 3 — Values of Student Test obtained for comparative analysis of the number of grains between the variety Renan wild-type (WT), for the individuals with one heterozygous copy (Aabbdd, aaBbdd, aabbDd) and for the triple mutant (aabbdd). Significant values (p-value < 0,05) are indicated in green. [file Table_3.docx]

Table S.3 : Values of Student Test obtained for comparative analysis of the number of grains between the variety Renan wild-type (WT), for the individuals with one heterozygous copy (Aabbdd, aaBbdd, aabbDd) and for the triple mutant (aabbdd). Significant values (p-value < 0,05) are indicated in green.

| **T. Test** | Aabbdd | aaBbdd | aabbDd | aabbdd |
| --- | --- | --- | --- | --- |
| Aabbdd |  |  |  |  |
| aaBbdd | 0,01559 |  |  |  |
| aabbDd | 0,44229 | 0,44118079 |  |  |
| aabbdd | 0,00011 | 0,14707111 | 0,06436236 |  |
| WT | 0,70017 | 0,01825556 | 0,50170038 | 0,00013709 |
