# Supplementary material for: TaRECQ4 contributes to maintain both homologous and homoeologous recombination during wheat meiosis
Source: Front Plant Sci. 2024 Jan 29;14:1342976. doi: 10.3389/fpls.2023.1342976 (PMC10859459; doi:10.3389/fpls.2023.1342976)
Supplement: Supplementary Table 4 — Percentage of cells with univalents, rod bivalents and multivalents for WT and each mutant for TaReCQ4. [file Table_4.docx]

Table S.4: Percentage of cells with univalents, rod bivalents and multivalents for WT and each mutant for *TaReCQ4*.

| **Number of cells counted** | **Genotype** | **% Univalent** | **% Rod bivalent** | **% Multivalent** |
| --- | --- | --- | --- | --- |
| 121 | WT | 0.0 | 28.1 | 0,0 |
| 190 | TM | 1.6 | 37.4 | 11.6 |
| 149 | Htz A | 0.0 | 40.9 | 2.0 |
| 144 | Htz B | 7.6 | 49.3 | 10.4 |
| 100 | Htz D | 5.0 | 39.0 | 5.0 |
