# Supplementary material for: TaRECQ4 contributes to maintain both homologous and homoeologous recombination during wheat meiosis
Source: Front Plant Sci. 2024 Jan 29;14:1342976. doi: 10.3389/fpls.2023.1342976 (PMC10859459; doi:10.3389/fpls.2023.1342976)
Supplement: Supplementary Table 5 — Mean and standard deviation of chiasma number and pairing forms in Renan wild-type (WT) and TaRecQ4 mutants. Counting was performed on 120 cells for each genotype. [file Table_5.docx]

Table S.5: Mean and standard deviation of chiasma number and pairing forms in Renan wild-type (WT) and TaRecQ4 mutants. Counting was performed on 120 cells for each genotype.

| **Mean** | **Chiasma** | **Univalent** | **Rod bivalent** | **Ring bivalent** | **Multivalent** |
| --- | --- | --- | --- | --- | --- |
| WT | 41,68 ± 0,103 | 0.00 | 0,32 ± 0,103 | 20,68 ± 0,103 | 0.00 |
| TM | 41,03 ± 0,340 | 0,02 ± 0,019 | 0,40 ± 0,134 | 20,20 ± 0,156 | 0,17 ± 0,089 |
| Htz-A | 41,51 ± 0,281 | 0.00 | 0,46 ± 0,322 | 20,48 ± 0,278 | 0,021 ± 0,012 |
| Htz-B | 40,54 ± 0,703 | 0,11 ± 0,046 | 0,75 ± 0,572 | 19,85 ± 0,672 | 0,135 ± 0,074 |
| Htz-D | 41,44 ± 0,469 | 0,09 ± 0,089 | 0,47 ± 0,423 | 20,40 ± 0,409 | 0,052 ± 0,008 |
