# Supplementary material for: TaRECQ4 contributes to maintain both homologous and homoeologous recombination during wheat meiosis
Source: Front Plant Sci. 2024 Jan 29;14:1342976. doi: 10.3389/fpls.2023.1342976 (PMC10859459; doi:10.3389/fpls.2023.1342976)
Supplement: Supplementary Table 6 — T. Test values obtained by comparative analysis of mutants between them and with WT for each pairing form category (rod bivalent, ring bivalent, multivalent) and no-pairing (univalent), p-value < 0.05. Boxes in green show the values for which the T test is significant. NA, not applicable. [file Table_6.docx]

Table S.6: T. Test values obtained by comparative analysis of mutants between them and with WT for each pairing form category (rod bivalent, ring bivalent, multivalent) and no-pairing (univalent), p-value < 0.05. Boxes in green show the values for which the T test is significant. NA: not applicable.
