# Supplementary figures and images for: TaRECQ4 contributes to maintain both homologous and homoeologous recombination during wheat meiosis
Source: Front Plant Sci. 2024 Jan 29;14:1342976. doi: 10.3389/fpls.2023.1342976 (PMC10859459; doi:10.3389/fpls.2023.1342976)

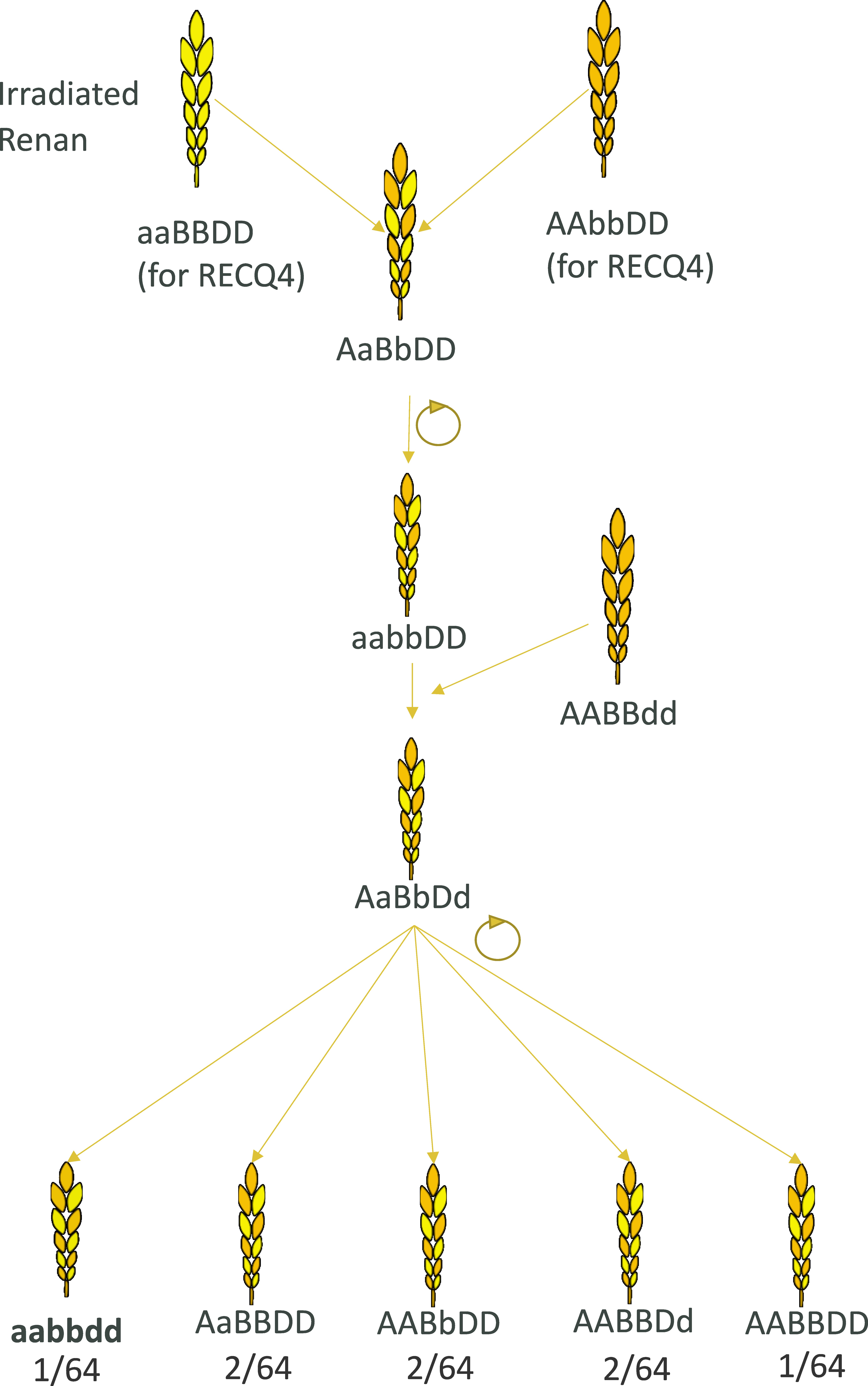

Supplement: Supplementary Figure 1 — Crossing scheme of wheat TaRecQ4 mutants to obtain a triple mutant (TM) and heterozygous mutants on one copy of one sub-genome (Htz-A, Htz-B, Htz-D). [file Image_1.jpg]

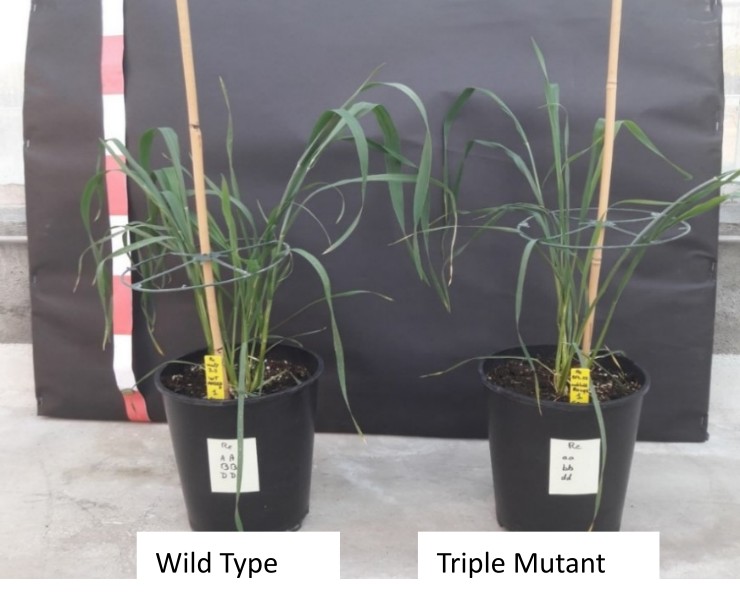

Supplement: Supplementary Figure 2 — Picture of Renan wild type on the left and the triple mutant for TaRecQ4 on the right. [file Image_2.jpg]

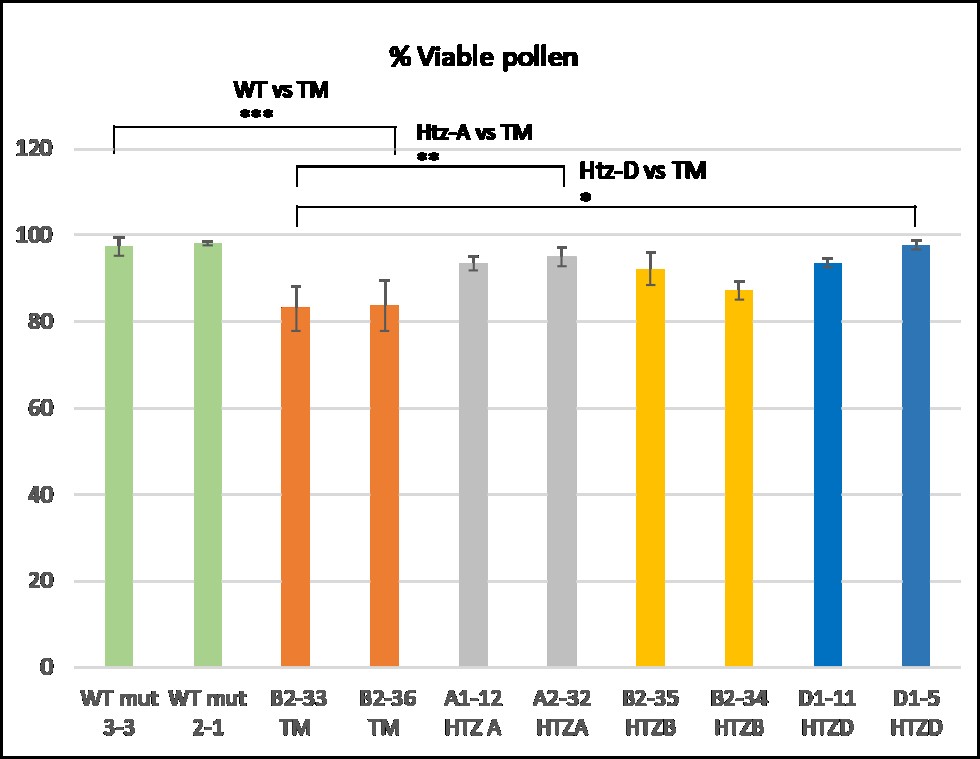

Supplement: Supplementary Figure 3 — Percentage of viable pollen for each category of mutants. T-Test was calculated and the significance was indicated by star(s): * p-value < 0.05; ** p-value p < 0.01; *** p-value < 0.001. [file Image_3.jpg]

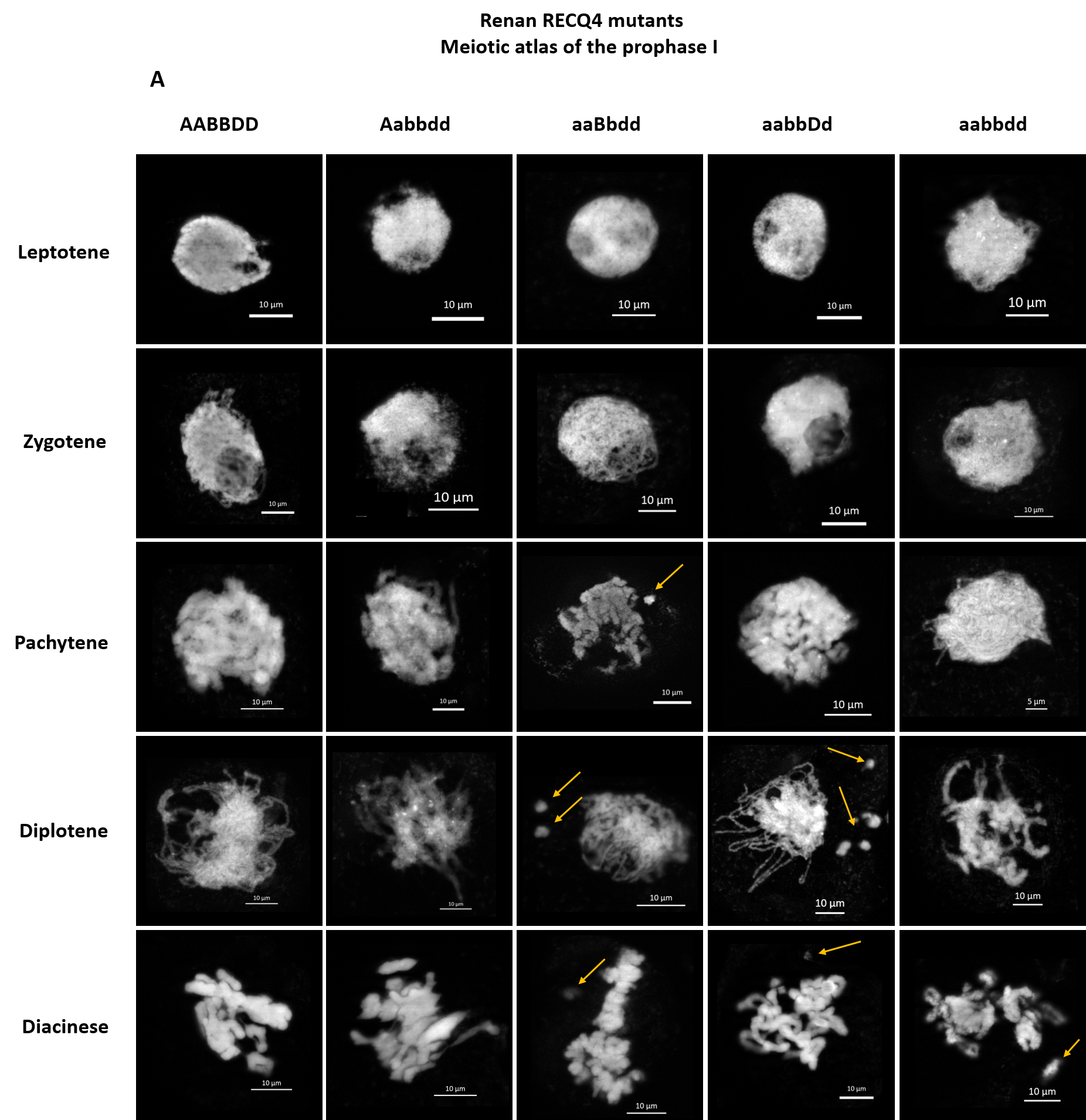

Supplement: Supplementary Figure 5 — Prophase I atlas from leptotene to diakinesis for WT, Heterozygous Htz-A, Htz-B, Htz-D and TM mutants. The orange arrows underline the fragmentations of the nucleus in the mutants. [file Image_5.tif]

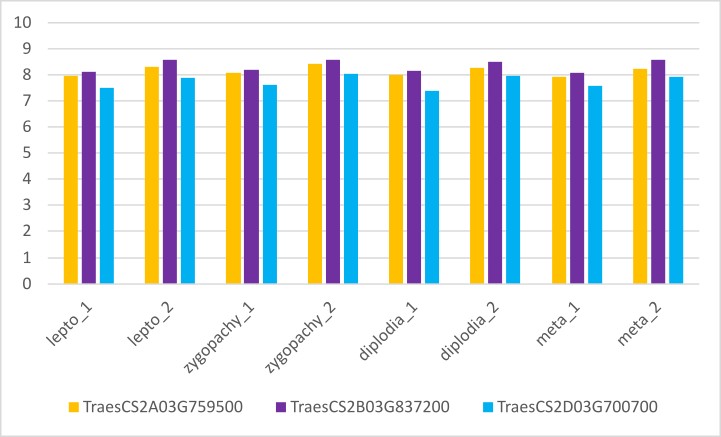

Supplement: Supplementary Figure 6 — level of expression of TaRecQ4 homoeologous copies using RNASeq data from Lloyd et al., 2014. Lepto_1 & _2: leptotene stage; zygopachy_1 & _2: zygotene and pachytene stages; diplodia_1 & _2: diplotene and diakinesis stages; meta_1 & _2: metaphase I stage. Two replicates (_1 & _2) are done for each stage. TaRecQ4 copies are colour-coded for the A- (yellow), B- (violet) or D (blue) genomes respectively. [file Image_6.jpg]

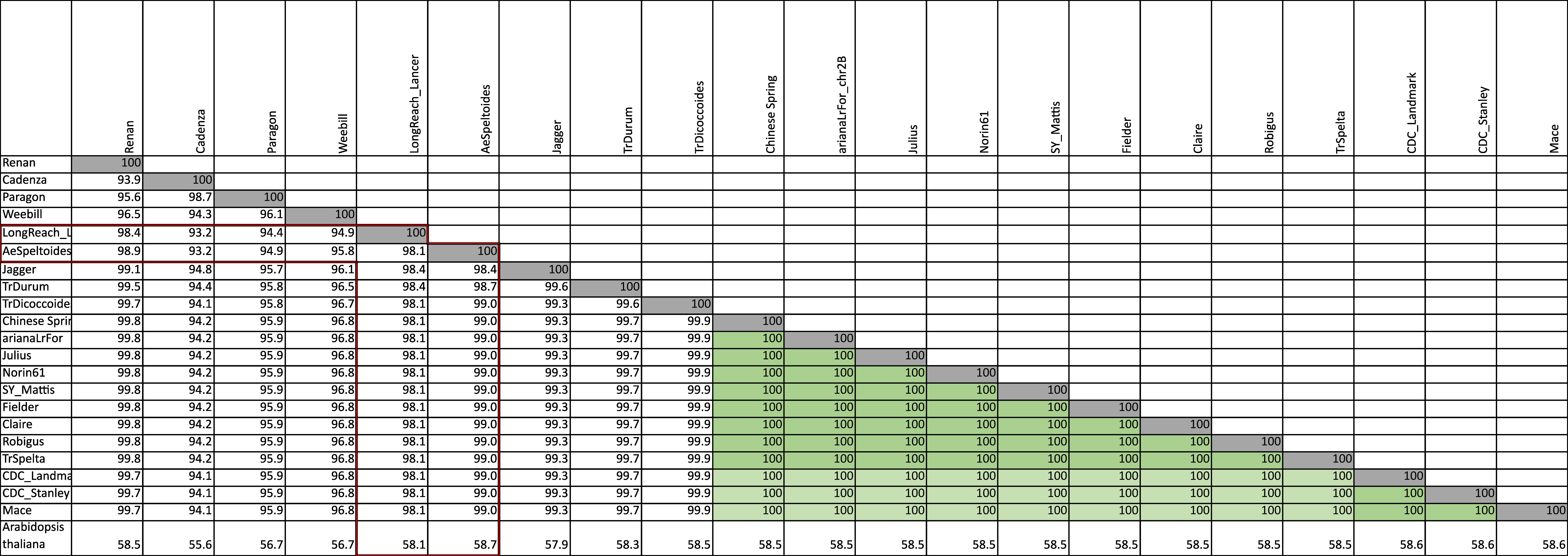

Supplement: Supplementary Figure 7 — Structure and motifs of TaRECQ4 protein from Renan found with Motif finder. [file Image_7.jpg]
