# Supplementary material for: TaRECQ4 contributes to maintain both homologous and homoeologous recombination during wheat meiosis
Source: Front Plant Sci. 2024 Jan 29;14:1342976. doi: 10.3389/fpls.2023.1342976 (PMC10859459; doi:10.3389/fpls.2023.1342976)
Supplement: Supplementary Figure 4 — Representation of OTVs and outliers, showing the deletion of the TaRECQ4 region on chromosomes 2A, 2B and 2D due to radiation. [file Image_4.pdf]

A

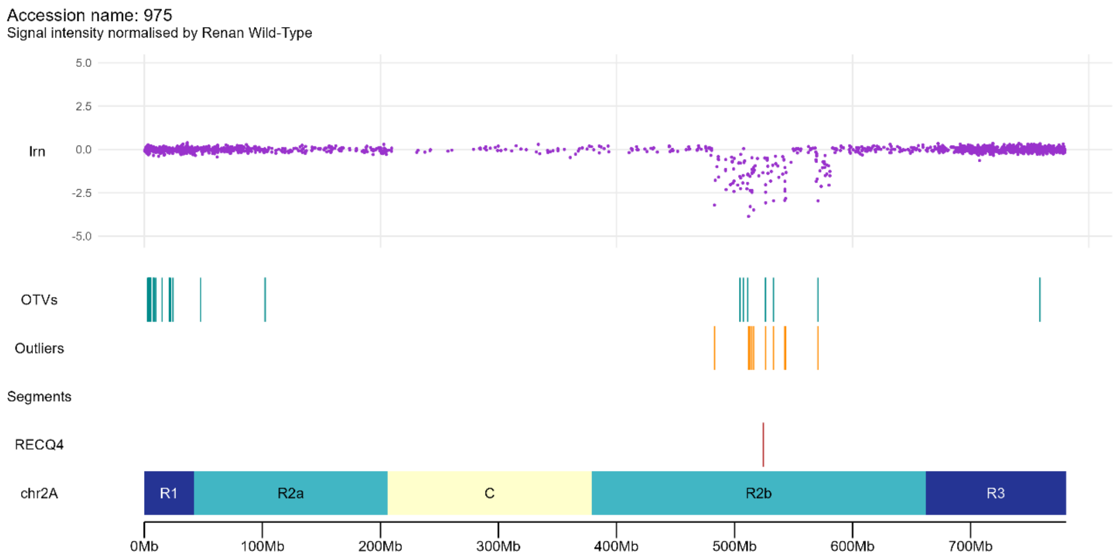
Accession name: 975
Signal intensity normalised by Renan Wild-Type
5.0
2.5
0.0
-2.5
-5.0
lrn
OTVs
Outliers
Segments
RECQ4
chr2A
R1
R2a
C
R2b
R3
0Mb
100Mb
200Mb
300Mb
400Mb
500Mb
600Mb
700Mb

B

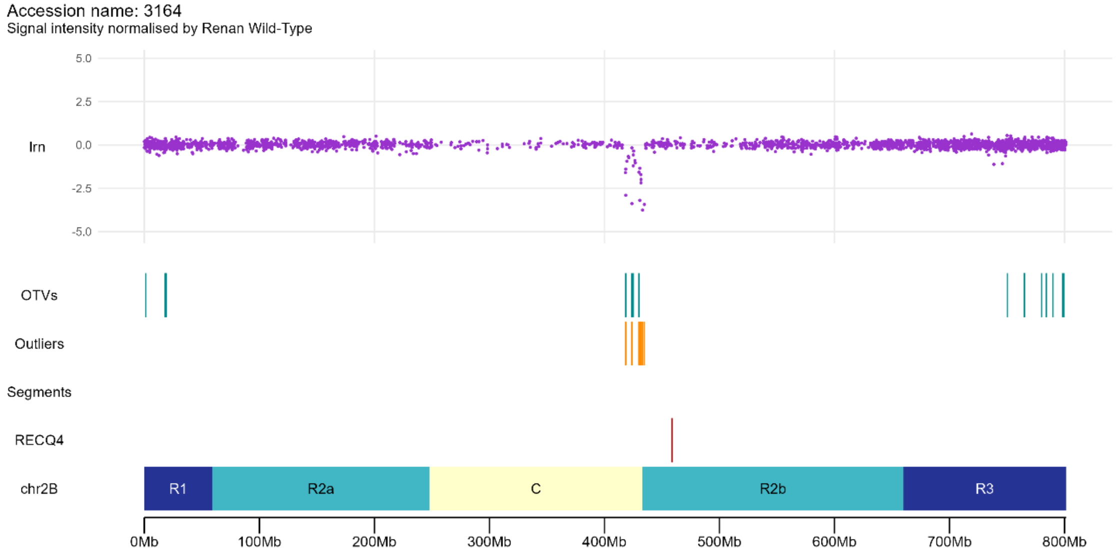
Accession name: 3164
Signal intensity normalised by Renan Wild-Type
5.0
2.5
0.0
-2.5
-5.0
lrn
OTVs
Outliers
Segments
RECQ4
chr2B
R1
R2a
C
R2b
R3
0Mb
100Mb
200Mb
300Mb
400Mb
500Mb
600Mb
700Mb
800Mb

D

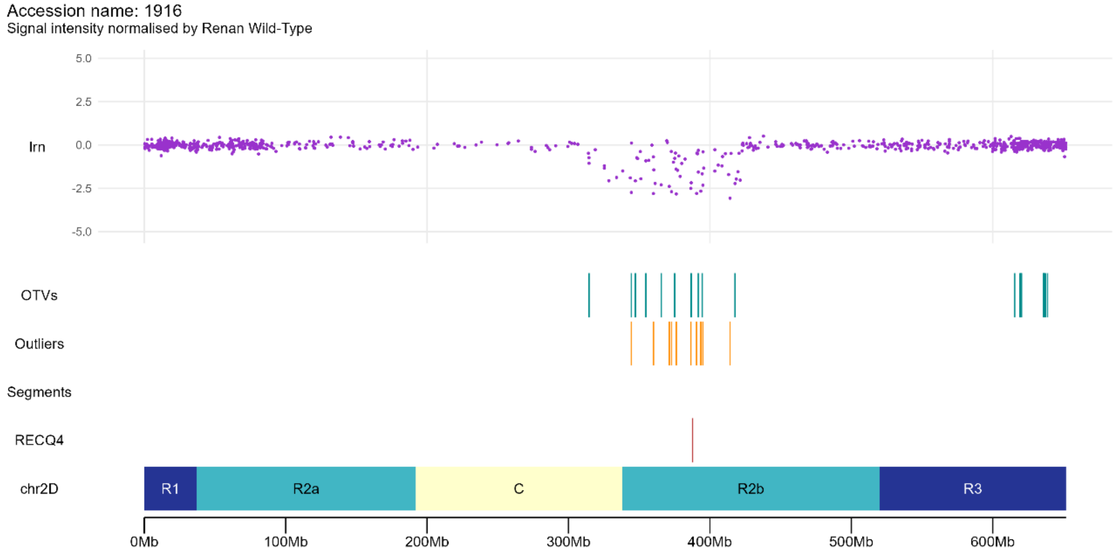
Accession name: 1916
Signal intensity normalised by Renan Wild-Type
5.0
2.5
0.0
-2.5
-5.0
lrn
OTVs
Outliers
Segments
RECQ4
chr2D
R1
R2a
C
R2b
R3
0Mb
100Mb
200Mb
300Mb
400Mb
500Mb
600Mb
